# Transformative Social Responses to Domestic, Family, and Sexualized Violence: A Qualitative Exploration of Insight Exchange, a Victim-Survivor-Centered Initiative Informed by Response-Based Practice

**DOI:** 10.1177/10778012231186812

**Published:** 2023-07-18

**Authors:** Leticia Funston, Ghena Krayem, Rita Shackel

**Affiliations:** 1 153403 Sydney Law School, 4334 University of Sydney, Sydney, New South Wales, Australia

**Keywords:** victim-survivor lived expertise, Response-Based Practice, domestic violence, family violence, social responses

## Abstract

This article reports on a qualitative study exploring victim-survivors’ and social responders’ experiences of Insight Exchange, an Australian-based victim-survivor-centered initiative informed by the center for response-based practice. This study involved 51 participants who completed an online survey (*N* = 43 social responders, *N* = 8 victim-survivors) and 16 participants (*N* = 12 social responders, *N* = 4 victim-survivors) who participated in semistructured interviews. The findings indicate that Insight Exchange has supported social responders to improve the quality of their responses to victim-survivors of violence. Victim-survivors reported on the value of Insight Exchange, which emphasized their resistance and responses to violence and abuse.

## Introduction

Insight Exchange (see website: https://www.insightexchange.net/) is an initiative of the Australian-based registered charity, Domestic Violence Service Management (DVSM) and has been funded through the support of individual private donations. Insight Exchange was designed and developed in collaboration with the Center for Response-Based Practice and primarily aims to improve the quality of social responses to victim-survivors of domestic, family, and sexualized violence through the development and distribution of freely available resources, knowledge translation, events, and research. Insight Exchange also centers victim-survivors’ lived expertise to provide “support to people who are experiencing domestic and family violence, and to inform people who are responding (formally and informally) in any community, service or system” (Insight Exchange, 2022). While there are many comparative initiatives in Australia and globally that aim to improve the quality of social responses to victim-survivors through knowledge translation and information sharing, the Insight Exchange initiative can be described as unique for a few reasons. Firstly, Insight Exchange focuses on applying, contextualizing, and developing Response-Based Practice theories and approaches for both formal and informal social responders. Insight Exchange provides direct and indirect support to responders to enhance their understanding and application of Response-Based Practice across diverse cultural, institutional, disciplinary, sectoral, and geographic contexts within Australia and internationally. The evidence base for Response-Based Practice is building in Australia and internationally indicating that this is a safe, accurate, and dignifying practice with victim-survivors experiencing multiple and intersecting forms of adversity such as, domestic and family violence, violence against children, sexualized violence, colonial violence, and racism (Alexander, 2022; Donovan et al., 2019; Hydén, Wade & Gadd, 2015; Richardson et al., 2021; Fast & Richardson Kinewesquao, 2019). Response-Based Practice also provides tools such as the *Interactional and Discursive View of Violence and Resistance, Four Operations of Language* (Coates & Wade, 2007) which can be applied to reveal and clarify accurate representations of violence (e.g., by clarifying perpetrator responsibility, honoring victim-survivors resistance to violence). The accurate representation of violence and resistance to violence is a central goal for the Insight Exchange initiative, as accurate representations of violence can improve the quality of social responses to victim-survivors and prevent victim blaming (Coates & Wade, 2007). The intensive focus and application of Response-Based Practice arguably set Insight Exchange apart from other initiatives that may utilize an eclectic theoretical base. Secondly, all information and reflective resources produced by Insight Exchange, centers and values the lived expertise of victim-survivors and particularly their insights concerning the quality of social responses they received from others and their resistance and responses to abuse and injustice. While victim-survivor lived expertise is increasingly recognized as an essential component of violence response and prevention work, academic and disciplinary knowledge continues to be privileged over the expert knowledge of victim-survivors (Domestic Violence Victoria and University of Melbourne, 2020). Insight Exchange is a leader in demonstrating the value of victim-survivors’ insights. Thirdly, the Insight Exchange initiative argues that responding to, preventing, and ultimately, eliminating the perpetration of domestic, family, and sexualized violence depends on the quality of social responses to victim-survivors, perpetrators, and other social responders (Insight Exchange, 2022). Most domestic and family violence resource development, research, education, and training focus on formal responders such as specialist domestic, family, and sexualized violence services and statutory and law enforcement services. While this focus is critically important, the work of Insight Exchange addresses a broader audience and is inclusive of all informal responders who interact with victim-survivors in diverse settings, for example, workplaces, friends and family members, businesses, education, community groups, health support services, helplines, lawyers, and academics.

This article reports on a qualitative study that explored victim-survivors’ and social responders’ experiences of the Insight Exchange initiative and reflects on the contribution of Insight Exchange in improving social responses in the context of responding to and preventing domestic, family, and sexualized violence.

### What Are Social Responses and Why Are They Significant in the Context of Domestic, Family, and Sexualized Violence?

Social responses can be understood as all behaviors and interactions including language, behavior, speech acts, body language, and written and visual representations (Coates & Wade, 2016). Social responses can be broadly categorized along a continuum ranging from complicit, harmful, and unhelpful, toward social responses that are supportive and helpful (Insight Exchange, 2022). Perpetrators of domestic, family, and sexualized violence frequently misrepresent their use of violence and abuse to “avoid accountability” (Wilson, 2022, p. 9). People who use violence also frequently misrepresent victim-survivors as “passive or even willing participants in the violence” and violence is minimized and excused as “nondeliberate” (Coates & Wade, 2007, p. 519). Perpetrators of violence and abuse have the most to gain from misrepresentations of violence and from harmful social responses which collude with the misrepresentation of violence. Whether harmful responses are intentionally or inadvertently complicit, they protect perpetrators and enable them to extend and escalate their use of violence, abuse, coercion, and control with adverse consequences for victim-survivors (Coates & Wade, 2007; NSW Domestic Violence Death Review Team, 2020). For these reasons, perpetrators often anticipate the type and quality of social responses they may receive, and the responses victim-survivors may receive from others (Coates & Wade, 2007). Perpetrators utilize these calculations in their deliberate efforts to extend abuse through families, communities, services, and systems (Coates & Wade, 2007).

In the context of responding to victim-survivors, complicit, harmful, and unhelpful social responses can include disbelieving the victim-survivor, maintaining a culture of institutional protectionism, minimizing the violence or abuse, and/or minimizing perpetrators’ responsibility, and pathologizing, victim-blaming, shaming, or criminalizing the victim-survivor (Coates & Wade, 2007; MacFarlane, 2020). Harmful social responses, that stigmatize, discredit, and devalue victim-survivors, can significantly undermine their safety, well-being, and physical and mental health (Overstreet et al., 2019, pp. 1734–1752; Sylaska & Edwards, 2014). Such misrepresentations can foster harmful social responses which “impede effective interventions through education, victim advocacy, reportage, law enforcement, criminal justice, child protection, and counselling with perpetrators and victims” (Coates & Wade, 2007, p. 521). Misrepresentations of domestic, family, and sexualized violence and harmful social responses can also compound and intersect with other forms of injustices including but not limited to institutional and systemic racism, sexism, ageism, ableism, classism, homophobia, and transphobia (Cramp & Zufferey, 2021).

Conversely, social responses can be used to honor victim-survivors’ resistance and responses to abuse, clarify perpetrators’ responsibility and expose violence (Coates & Wade, 2007). When victim-survivors receive positive and affirming social responses they are more likely to talk about what has happened or is happening to them, to formally report the violence and continue to engage with services that support their health, well-being, and advocacy (Laing & Humphreys, 2013). Supportive social responses hold perpetrators to account for their actions and convey to others that the perpetration of domestic, family, and sexualized violence and abuse will not be tolerated or excused (Coates & Wade, 2007). Supportive social responses help reveal the perpetrators’ intentionality, choices, and decision-making to use violence. Furthermore, supportive social responses can also challenge the pervasive social discourse that perpetrators are not in control of and not responsible for their behaviors (Coates & Wade, 2007). Victim-survivors also report a strong preference for social responses that are nonjudgmental, holistic, validating, and unconditionally accepting of the victim-survivor, in order to meet their safety and well-being needs (Allen, Larsen, Trotter & Sullivan, 2013).

### Insight Exchange: A Collaborative Initiative With the Center for Response-Based Practice

Insight Exchange promotes the lived experiences and expertise of victim-survivors through an “Insight component” and an “Exchange component.” For the “Insight component,” Insight Exchange invites victim-survivors to voluntarily share their deidentified lived experience through a semistructured interview with Insight Exchange staff who are skilled in Response-Based Practice interviewing. For example, during the interview, Insight Exchange staff ask victim-survivors questions focused on their responses and resistance to the violence and abuse used against them (Coates & Wade, 2007). The interview questions also focus on the quality of the social responses received by the victim-survivors (Coates & Wade, 2007). In consultation with the victim-survivor, the interview transcript is de-identified, assembled, and edited into a cohesive narrative. With the victim-survivor's consent, their deidentified narrative is published on the Insight Exchange website.

The “Exchange component” involves sharing the de-identified lived experience narratives and insights of victim-survivors with other victim-survivors and informal and formal social responders. The Exchange component also informs social responders of their responsibility in providing supportive and helpful social responses to victim-survivors. Direct quotes from the victim-survivor narratives are centered in all Insight Exchange domestic, family, and sexualized violence knowledge translation resources, and seminars and events. Insight Exchange electronic resources are freely available on the Insight Exchange website.

Insight Exchange applies Response-Based Practice theoretical and practice-based standpoints in all aspects of the initiative including in the development and distribution of resources, during consultations with victim-survivors of domestic, family, and sexualized violence, and with social responders. For this reason, it is important to briefly introduce here the work of response-based practice. Response-Based Practice can be used as a counseling modality, research methodology, and method of analysis (Coates & Wade, 2007). According to the founders of Response-Based Practice, the key ideas “arose from direct service with people who had endured violence including Indigenous women and men who were violated in the so-called Residential Schools/Canadian Prison Camps” (Richardson & Wade, 2015, p.205). Response-Based Practice theories are also informed by critical realism, research methodologies, education, supervision, and advocacy approaches (Coates & Wade, 2007).

## The Current Study Aims

As accurate representations of violence and supportive social responses are essential components in responding to and preventing domestic, family, and sexualized violence, the authors chose to study the Insight Exchange initiative given its unique and distinctive focus on these specific approaches. This study aimed to explore participants’ experiences of Insight Exchange to identify some of the perceived benefits for victim-survivors and social responders and to identify the limitations of the initiative. This study considered the following research questions: (1) what are victim-survivors’ (with lived experience of domestic, family, and sexualized violence who shared their deidentified narratives with Insight Exchange) experiences of Insight Exchange? (2) What are social responders’ (who are engaged in social responses to domestic, family, and sexualized violence) experiences of Insight Exchange? These broad research questions aimed to explore how the Insight Exchange initiative may have contributed to the understanding and quality of social responses from participants who identify as social responders. The research questions also aimed to explore how the social responses from Insight Exchange were experienced by victim-survivors.

## Method

This research project was funded by a University of Sydney Law Engagement Partnership Project Grant to encourage collaboration and partnership between researchers and industry/community partners. This research project received formal ethics approval (Project No: 2020/667) from the University of Sydney Human Research Ethics Committee. The research project primarily utilized a qualitative research methodology, triangulated by quantitative survey data, in two phases. Phase 1 consisted of two online web-based surveys powered by Qualtrics. One survey was developed for victim-survivors and the other was developed for social responders. Both surveys utilized a combination of qualitative response options, multiple choice, and Likert scale questions. Upon completion of the survey, participants from both cohorts were invited to participate in the second phase of the study. Phase 2 involved semistructured interviews with victim-survivors of domestic, family, and sexualized violence (who had shared their lived experience deidentified narrative with Insight Exchange) and social responders. At no stage of the study were participants asked to explicitly describe or identify any experiences of domestic, family, and sexualized violence. The researchers did not ask participants to disclose types of violence and abuse experienced as this may have contributed to participant distress and raised ethical concerns and this focus did not address the research questions.

### Recruitment Process

In this study, the term social responder is used to refer to any person who intentionally or unintentionally responds to a victim-survivor of domestic, family, and sexualized violence. By this definition, every person is potentially a social responder to violence and abuse, whether they are aware of it or not. Social responders may include any person from any disciplinary or industry background including, the media, cultural and arts-based industries, government, nongovernment, and corporate organizations as well as police, legal staff, child protection, health workers, and domestic and family violence frontline workers. Social responders may also include work colleagues, friends, family, and community members and/or other acquaintances. The recruitment for Phase 1 commenced in July 2021 and concluded in October 2021. Recruitment for Phase 2 commenced in August 2021 and also concluded in October 2021. Participants were recruited into this study through a targeted email advertisement which was received by all people who had voluntarily signed up to the Insight Exchange mailing list. Participants were also recruited into this research project through an advertisement, including participant information, which was hosted on the Insight Exchange website. All participants who identified as social responders and who indicated that they had some familiarity with the work and ideas of Insight Exchange were invited to participate in the survey and the in-depth interview. However, the following inclusion criteria were used to ensure that participants had a sufficient level of familiarity with Insight Exchange: (a) social responders who have read or used at least one Insight Exchange resource, and/or (b) participated in at least one Insight Exchange event, and (c) signed up to the Insight Exchange mailing list for longer than 3 weeks. Social responders who did not meet the criteria were excluded from the study due to their limited engagement and familiarity with Insight Exchange and its resources. To minimize any real or perceived conflict of interest, people who govern or are employed/contracted by Insight Exchange were excluded from this study. These people included the DVSM Board and DVSM employees as Insight Exchange is an initiative of DVSM and Insight Exchange’s former and current Associates (as of June 2021).

In this study, some social responder participants also identified as victim-survivors of domestic, family, and sexualized violence, however as they had not shared their lived experience narrative with Insight Exchange for the purposes of this research project they were categorized as “social responders”. These participants were invited to complete the social responder-focused survey and the social responder semistructured interview. The category “victim-survivor” was reserved for participants who had previously shared with Insight Exchange, their deidentified narrative detailing their lived experiences, resistance, and responses to domestic, family, and sexualized violence. Victim-survivors were recruited into this study through a targeted email for people who had previously engaged in the Insight Exchange lived experience narrative. Victim-survivors who had not previously shared their lived experiences with Insight Exchange through this process were excluded from the study.

### Phase 1: Online Survey

Participants were initially invited to complete an online survey as either (a) social responder or (b) victim-survivor. Both online surveys included an invitation for participants to submit their contact details for participation in the second phase of the research, the semistructured interviews. All participants who provided their contact details were individually contacted by the researcher. Of the participants who identified as social responders, one participant declined the interview due to challenging personal circumstances, one participant did not meet the inclusion criteria due to being on the Insight Exchange mailing list for less than 3 weeks and two participants were contacted but did not reply. Of the participants who identified as victim-survivors, all participants (*N* = 5) who provided contact details participated in the semistructured interview with the exception of one participant who was contacted but did not reply. A brief overview of the survey participants’ demographic data is presented in Table 1. A total of 43 social responders and eight victim-survivors completed the online survey.

**Table 1.** Semi-Structured Interview Participants Brief Biographic and Demographic Information.

Interviewee 1 (social responder): is a child, youth, and family project worker who facilitates domestic and family violence prevention training for faith leaders.
Interviewee 2 (social responder): is a domestic and family violence counselor at a women's service.
Interviewee 3 (social responder): is a domestic and family violence worker and facilitates training sessions with staff.
Interviewee 4 (social responder): is a diversity and inclusion officer at a higher education institution.
Interviewee 5 (victim-survivor and published a lived experience narrative with Insight Exchange): disclosed lived experiences of domestic, family, and sexualized violence as an adult. Interviewee 5 also works in human resources in the entertainment industry and is a social responder.
Interviewee 6 (victim-survivor and published a lived experience narrative with Insight Exchange): disclosed lived experiences of domestic, family, and sexualized violence as a child and adult.
Interviewee 7 (victim-survivor and published a lived experience narrative with Insight Exchange): disclosed experiences of domestic, family, and sexualized violence as an adult. Interviewee 7 is also a retired educator.
Interviewee 8 (social responder): works in government policy specializing in the delivery of services to victim-survivors of domestic and family violence. Interviewee 8 also disclosed having lived experience of domestic and family violence as a child.
Interviewee 9 (social responder): is a senior clinical manager of a counseling service specializing in domestic, family, and sexual assault. Interviewee 9 also disclosed having lived experiences of domestic, family, and sexualized violence.
Interviewee 10 (social responder): is a psychologist working in a national violence prevention organization. Interviewee 10 also disclosed having lived experiences of domestic and family violence.
Interviewee 11 (social responder): has a background in leadership, executive coaching, and has been working at a regional domestic and family violence service for over 3 years.
Interviewee 12 (social responder): is a medical director of the sexual assault service at a major Australian metropolitan hospital and specializes in medical forensic examinations. Interviewee 12 also facilitates statewide training for doctors and nurses who specializing in medical forensic work.
Interviewee 13 (social responder): is a social worker with over 25+ years of experience who supervisors’ counselors who work at a national domestic and family violence telephone counseling helpline service.
Interviewee 14 (social responder): has a background working with child and adult victims of violence and currently works with women involved in the criminal justice system.
Interviewee 15 (victim-survivor and published a lived experience narrative with Insight Exchange): is a single mother and has lived experiences of domestic and family violence. Interviewee 15 is also a social responder who has worked in the domestic, family, and sexualized violence sector.
Interviewee 16 (social responder): has worked in the domestic, family, and sexualized violence sector for the past 3 years primarily in men's behavior change. Interviewee 16 also disclosed having lived experiences of domestic, family, and sexualized violence.

### Phase 2: Semistructured Interview

Due to the COVID-19 pandemic measures at the time of the study, all semistructured interviews were conducted online via video conferencing platforms or via telephone. Please see Appendix A for the semistructured interview questions for social responders and Appendix B for victim-survivor participants. Interviews were approximately 50 min in duration and were audio-recorded on a digital recording device and transcribed. Additional steps were incorporated into the study design to anticipate and respond to potential participant distress. To reduce the potential for distress, the questions in the survey and semistructured interview centered specifically on participants’ experiences of engaging with the Insight Exchange resources. Participants were not asked any direct or indirect questions about their lived experiences of domestic, family, and sexualized violence. All participants were provided with contact details of freely available support services to support any participants who may have experienced distress related to participating in the survey or semistructured interview. Participants who completed the semistructured interview also received a follow-up email and/or text message from the researchers and were asked if they required further support. Some participants voluntarily shared that they have lived experiences of domestic, family, and sexualized violence. However, no participants reported feeling distressed during or following the semistructured interviews. To protect the identities of all participants, the term “interviewees” has been used throughout.

A total of twelve social responders and four victim-survivors completed the semistructured interview. See Table 2 for additional contextual and demographic information about each of the participants who to took part in the semistructured interviews. Participants were encouraged to share any demographic details they wanted readers to know about and were also encouraged to choose to conceal any information.

**Table 2.** Survey Participant Demographics.

	Victim-survivors (total number)	Social responders (total number)
**Gender Identity**		
Nonbinary	1	1
Female	7	33
Male	0	4
**Identity**		
Aboriginal	0	0
Torres Strait Islander	0	0
Non-Aboriginal	8	38
Culturally and Linguistically Diverse	0	7
**Age**		
18–24	0	0
25–34	0	1
35–44	4	14
45–54	2	13
55–65	0	9
65+ years	2	1
**Religious/spiritual identity**		
Muslim	0	1
Christian	0	4
Catholic	0	2
Anglican	0	1
Baptist	0	1
Jewish	0	1
Buddhist	0	0
Agnostic/spiritual but not religious	1	17
Atheist	2	6
**Work**		
Full-time	6	22
Part-time	0	13
Casual	0	1
Unemployed	2	2
Student	0	0
**Education**		
High school (Yr 10 or equivalent)	1	0
High school (Yr 12 or equivalent)	3	5
Undergraduate degree	1	7
Postgraduate degree	2	24
Doctorate	1	2
**Location**		
New South Wales	3	25
Australian Capital Territory	0	0
Victoria	1	3
Tasmania	0	0
South Australia	0	2
Western Australia	0	0
Northern Territory	0	0
Queensland	0	3

### Analysis

This study utilized both method triangulation and investigator triangulation to analyze the data to: (a) minimize the potential for bias, (b) develop a thorough understanding of the data from multiple perspectives, and (c) as a strategy to “test validity through the convergence of information from different sources” (Carter et al., 2014, p. 545). This study primarily collected qualitative data through semistructured interviews and qualitative survey questions. We used simple statistics from the survey data to further interrogate the qualitative analysis; the use of multiple methods of analysis is called method triangulation (Fetters et al., 2013). The use of multiple investigators in the analysis of the qualitative data is a form of investigator triangulation (Santos et al., 2020).

The qualitative data from the surveys and the semistructured interviews were analyzed in three stages. During the first stage of analysis, all three authors independently reviewed each interview transcript and anonymous qualitative survey data and developed descriptive categories. The authors then reviewed and discussed the descriptive categories identified by each author. The authors then decided on seven main descriptive categories. These descriptive categories were analyzed during a second stage of review, which minimized the methodological problem of producing purely descriptive categories, rather than analytical higher-order themes (Bazeley, 2009). Finally, the qualitative data were assessed for outliers, participants who had very divergent views in comparison with other participants. This also helped test both lower-order and higher-order themes during the third and final stage of analysis (Bazeley, 2009). This led to the construction of seven higher-order themes. The quantitative data from the surveys were first analyzed using basic statistics and were then used to test the seven higher-order themes through triangulation (Bazeley, 2012; Carter et al., 2014). For instance, the quantitative data showed overall that following engagement with Insight Exchange, social responders reported having increased knowledge and understanding of domestic, family, and sexualized violence overall, improved understanding of victim-survivors’ experiences, and improved knowledge of perpetrator behaviors. These quantitative data supported the themes identified by the investigators. Following this analysis, the investigators agreed on the themes presented in the findings section.

## Findings

In the attempt to increase transparency in presenting the findings section, we have used the following guide developed by Rawsthorne (2009, p. 49) and Tseris (2014, p. 92): The terms “most” or “the majority” were used to refer to at least 75% of participants; the terms “many” or “several” or “a number of” were used to refer to at least half (50%) of the participants; the term “some” was applied to refer to more than three participants but less than half of the participants; and the terms “a few” or “a small number” were used to refer to less than three participants.

### The Importance of Accurate Representations of Violence and the Language of Resistance (Informed by Insight Exchange)

Social responder participants reported that Insight Exchange had improved their understanding of domestic, family, and sexualized violence through the clear representations of violence and the consistent use of language emphasizing victim-survivors’ responses and resistance to domestic, family, and sexualized violence. The qualitative data from the surveys and semistructured interviews suggests that Insight Exchange supported social responders to challenge dominant victim-blaming and shaming discourses in their practice. Interviewee 10 reflected on the subtle yet meaningful difference between working from Insight Exchange/Response-Based Practice approaches and the strengths-based approaches, which are widely used in human and health services:

I hear it in our language, and I will sometimes need to correct it and say ‘no, you’re not talking about their (victim-survivors’) strengths, you’re talking about action’. It is not qualities of self, it is just actions they have taken and that is distinctive and that is quite different. (Interviewee 10, social responder)

While well intended, the use of strengths-based social responses alone may not always be appropriate in responding to victim-survivors of domestic, family, and sexualized violence. The strengths-based approach emphasizes socially acceptable efforts and actions and can be misused by social responders. This can diminish the identification and acknowledgment of victim-survivors’ acts of resistance and responses to violence—especially if victim-survivors’ responses are not socially palatable or acceptable. For example, many victim-survivors use alcohol and other drugs (Noonan et al., 2017), and self-harming such as cutting, as ways of responding to, and resisting violence and abuse (Ursa & Koehn, 2015). Some victim-survivors may have had the experience of perpetrators’ using praise and recognition as tactics to gain access to and manipulate the victim. As such, many victim-survivors may feel suspicious of the use of arbitrary "strengths-based" praise from social responders. However, the focus on a person's responses and resistance to violence using their own language and narrative safely conveys respect while giving space to the victim-survivor to name and reflect on their own insights and capacities.

Interviewee 2 also described the limitations of the strengths-based approaches and expressed a preference for working from the Insight Exchange/Response-Based Practice approach of asking victim-survivors about their acts of resistance, their responses, and decision-making during counseling sessions at a domestic and family violence service:

The language I feel has changed from a more empowering and less patronizing thing … Phrases I use a lot is ‘explicit choice’, ‘conscious decision’, ‘active choice’. I will use words like that about both the person who uses violence in their behavior, but also the women I work with in their behavior. To really reframe what has been happening as choices and decisions and exercises of power and being deliberate versus he was violent, and you survived and ‘good on you for surviving’. (Interview 2, social responder)

Most social responders described witnessing positive changes in the victim-survivors they worked with as a result of using language focused on victim-survivors’ responses and resistance to violence. For example, Interviewee 11 said that she has witnessed many victim-survivors express relief and profoundly positive emotional responses once their resistance to violence has been revealed, sometimes as early as during the initial intake assessment:

There is such healing when we use that language (of resistance) in the intake. The women just sob and sob. Because it is the first time where they (acknowledged), they have been doing something. That sense of there is a way out, there is something that I can do, and I have been doing it, and I have not just let this happen. Because the social narrative is still mistrust, disbelief, and tell survivors what to do. (Interviewee 11, social responder)

Similarly, Interviewee 2, gave an example of working with a victim-survivor who held shame-based beliefs about herself. Interviewee 2 described how her attention to the victim-survivor's decision-making and choices helped to highlight the victim-survivor's inherent capacities, which disrupted the abusive narratives she held about herself:

Using Response-Based Practice interviewing really proved to her that she can trust herself, that she is an adult and she is not stupid. Those are words she used to describe herself; ‘stupid’ and ‘old’ and ‘silly’ and ‘how do I do all that sort of stuff’. And yet we have this proof that she made choices. She never thought of it that way (before). (Interviewee 2, social responder)

Other social responders felt that the language of victim-survivors’ resistance and responses to violence increased victim-survivors’ confidence in their own capacities and quality of life:

We see much more confidence (in victim-survivors) …. For the first time ever, they feel confident. They are able to get out in public, walk in community, do things. I have seen women being drug and alcohol-free for 4 years now and we are talking about women who have had their dignity, their self-esteem stripped from victimization. So, to have that kind of shift is huge. (Interviewee 14, social responder)

Interviewees 13 and 14 also felt that Insight Exchange facilitated a deep understanding of victim-survivors’ responses that are not commonly viewed as “resistance.” For example, one social responder reflected, “(learning) that resistance comes in all forms …. My eyes were opened” (Interviewee 13, social responder). Interviewee 4, also described how she had no prior learning or training in exploring the diversity of victim-survivors’ responses and resistance to violence and abuse:

Allan Wade (Center for Response-Based Practice) spoke (at the Insight Exchange event Creating Conversations) about the resistance to violence, and that was something I probably was not aware of. I was not aware of the full subtlety of (resistance) as well in terms of a response to violence …. Victims’ resistance and response to violence had not been highlighted anywhere else. (Interviewee 4, social responder)

Other social responders gave examples of how they have come to understand victim-survivors’ decisions not to act and their efforts to “appease” perpetrators through the lens of their resistance.

Resistance can be about not saying anything and not doing something. It is about the importance of not making any assumptions (Interviewee 13, social responder).

It gave voice to the decision not to speak up. It gave voice to the decision for ‘I'm going to play along with this to keep myself safe as I can be right now’ … I think the resistance of responses is huge for me. Because it gave voice to a whole spectrum of behaviors and actions which have historically been either silenced or negatively viewed or put out for some reason as not being a valid response…. Where in the past they were either put down for not responding and just being passive or being aggressive and fighting or for using substances and not taking that substance use as a form of escapism or a form of survival. (Interviewee 14, social responder)

Most social responders (*N* = 18) who completed the online survey either agreed or strongly agreed that Insight Exchange had contributed to their understanding of the behaviors of perpetrators of domestic, family, and sexualized violence. Most social responder participants said that they valued Insight Exchange's representation of the perpetration of domestic, family, and sexualized violence as a deliberate and active choice. Social responders said that Insight Exchange's messaging helps to counteract the prevalent misrepresentation of the perpetration of domestic, family, and sexualized violence as an “anger management issue”. The “anger management” discourse arguably misrepresents acts of violence as explosive and opportunistic rather than premeditated and tactical: “Insight Exchange helped me to understand there's a lot of choice and control around where, when and how they (perpetrators of domestic violence) exhibit their controlling, coercive, abusive behavior” (Interviewee 9, social responder). The focus on perpetrators’ choice in using violence and abuse also facilitated a socio-political analysis of domestic and family violence for some social responders:

I can see how patriarchal layers, social system, and all of that, is contributing to, I guess, perpetrator's beliefs about (women). That men somehow have more rights than women and the right to exert their power over others. (Interviewee 9, social responder)

Similarly, Interviewee 4 said that since engaging with Insight Exchange her organization now uses the term, “person using violence” to emphasize the deliberate use of violence and abuse:

What was really important to those with lived experience in our organization was the message that (the perpetrator) was in control. They were not out of control, it was deliberate. (Interviewee 4, social responder)

Response-Based Practice (Coates & Wade, 2004) and Insight Exchange also encourage social responders to clarify the perpetrator responsibility and shift away from the question; “why doesn’t she just leave?” to “what is he (the perpetrator) doing to undermine the victim-survivor's safety and well-being and to oppress her resistance and responses to his violence?” Social responders commented on the significance of this distinction:

We still see the residue of why ‘does not she leave’. Mutualizing responsibility and mutualizing the violence. Just because (victim-survivors) could not stop the violence does not mean they let it happen. So, we try and have those more nuanced conversations, but to ‘why does not she leave?’ we say, ‘What do you think, why wouldn'tt she?’ So, I think Insight Exchange gives us language to provide more nuance. (Interviewee 11, social responder)

Interviewee 11 also described how Insight Exchange resources are used in the domestic and family violence service's certified Men's Behavior Change Program. This service utilized Insight Exchange resources to support staff (during their internal Men's Behavior Change Program training), and in direct practice with men who are asked to consider the deliberateness of their own actions and alongside recognizing the resistance and responses of victim-survivors during their interactions. For Interviewee 10, the Insight Exchange resource *I Am, I Can* “beautifully and simply and articulately highlights that using violence is a choice.” For interviewee 10 and other social responders, framing the perpetration of domestic, family, and sexualized as a choice is “in the interest of the user of violence because that means that they can choose differently.” Interviewee 10 further explained that the language of perpetrator's choice increased perpetrators’ engagement in programs such as Men's Behavior Change and facilitated greater accountability for the decision to use violence and abuse:

We are always trying to balance the need to challenge users of violence and hold them accountable without losing their engagement. We do not want to collude, but we do not want to challenge them too much so that they check out and that is a fine line…. Violence is a choice, but you can choose to be nonviolent and here are some pathways to do that or if you want to kind of start heading down that track. I think that is probably why I felt like (Insight Exchange) had the engagement balance right and did not do too much challenging, but also did not kind of collude either. (Interviewee 10, social responder)

### Victim-Survivors Revealed Their Resistance and Responses to Domestic, Family, and Sexualized Violence

For many victim-survivor participants, speaking with Insight Exchange was the first time their experiences of domestic, family, and sexualized violence had been explored with an emphasis on their resistance and responses to violence and abuse. Most victim-survivors described Insight Exchange's focus on their resistance as “life-changing” as many victim-survivors came to understand their actions and responses were aimed at increasing safety and dignity for themselves and others, even if they were unable to stop the violence. This shift in understanding supported many victim-survivors to let go of victim/blaming narratives and shame. For Interviewee 15, Insight Exchange helped her to resist unhelpful mental health labels which were imposed on her by staff at a domestic and family violence service; “When I was in the refuge, I was told that I had PTSD, and by the time I’d finished with Insight Exchange, I was like, ‘no, no, no, I was just responding to violence, I’m good, you’ve got the problem’.” Interviewee 15 went on to describe how she experienced speaking with Insight Exchange staff about her lived experiences and the meaning of being asked strategic questions focused on her resistance and responses to violence and abuse:

It was (an Insight Exchange interviewer, the Insight Exchange director, and the associate director) all together, and they were like, ‘no, this is a response to violence, and it is how you responded to someone being violent toward you’, which changed my entire perspective on myself … it was not someone telling me ‘you are this and do not worry, it is not your fault’. It was not like that. They showed you that it was not your fault. By your own examples, they would show you about your resistance. They explained that in a way that was so personalized … I was able to explain how I would self-monitor. I would base my day and the way that I interacted upon what tone he had when he came home, how he spoke to me on the phone, and I would change according to that. I would be very vigilant in monitoring everything …. All those little things that contributed to why I did what I did. And I never actually even explored that myself. They actually taught me or helped me reconstruct my idea of the past and the idea of myself in such a different, more positive way than all the guilt and all the pathologizing … (Interviewee 15, victim-survivor)

Similarly, for Interviewee 5, engaging with Insight Exchange led to “life-changing” realizations about her responses and resistance to the violence she survived, which engendered a sense of trust in herself:

Follow My Lead (Insight Exchange resource) for me was a turning point because I had never received help from any organization, I literally just left (ex-partner) cold turkey. So, no one knew about the violence or anything. Because it was guilt, right? There was guilt for me not having left earlier and having spent 13 years. But reading Follow My Lead I realized it was not safe for me to leave at that time. So, there was this real kind of awakening and trust in myself and what I was doing. (Interviewee 5, victim-survivor)

Interviewee 6 shared that speaking with Insight Exchange about her lived experiences, helped release long-held feelings of shame and self-blame. Interviewee 6 said that it was especially meaningful when an Insight Exchange Interviewer had rephrased her words about her experiences of sexualized violence from “my dirty little story” to “it is not your dirty story, it is their (the perpetrator's) dirty story.” This rephrasing served to clarify the perpetrators’ responsibility for the violence and abuse which helped Interviewee 6 to let go of the long-held belief that the sexualized violence perpetrated against her was her fault. Interviewee 6 also described how the Insight Exchange interview helped her to identify her many acts of resistance to violence and abuse. Interviewee 6 said this helped her to reveal her sense of personal “agency,” “voice,” and prompted her to engage in domestic, family, and sexualized violence prevention activism:

Insight Exchange started that journey because my doctor has been saying for years about the importance of having agency and I had no idea what agency was, and I did not know what it felt like. And then I went to Insight Exchange twice to tell my story, and then I got this feeling … I could really feel the agency. This happened after the first time I spoke with Insight Exchange, it was a bit of a mood lifter …. Some days I’m so stuck in depression that I couldn’t be bothered getting out of bed. But agency is a mood lifter. Agency gives you purpose. So, I only felt that when I went to Insight Exchange.

Similarly, an anonymous participant in the victim-survivor online survey commented that engaging with Insight Exchange helped the participant to critically interrogate blame and shame:

It helped me cement that there is not something wrong with me because I experienced revictimization. But that there are so many men out there abusing women from all backgrounds.

### Social Responders With Lived Experiences and Victim-Survivors Who Become Social Responders

Many social responders who participated in the online survey (*N* = 13) reported having lived experiences of domestic, family, and sexualized violence as an adult, and (*N* = 10) reported lived experiences of domestic, family, and sexualized violence as a child. Similarly, some social responders (*N* = 5) who participated in the semistructured interview said that they had lived experiences of domestic, family, and sexualized violence as a child or adult or both. All victim-survivor participants said that they were currently working or volunteering as social responders to domestic, family, and sexualized violence. These findings are meaningful in that they suggest that the distinction between victim-survivor and social responder is not a binary opposition as it is sometimes represented (Wilson & Goodman, 2021).

Many social responders with lived experiences of domestic, family, and sexualized violence shared that Insight Exchange's explicit use of victim-survivors’ voices and lived experiences in resources and initiatives increased their trust and confidence in the ideas:

I do have lived experience as many people do working in this sector. I think, that when I read the Insight Exchange resources, wearing two different hats at the same time is actually quite useful. I think I probably connect more with the Insight Exchange resources or have more faith and trust in them, because, you know, I can identify with them. (Interviewee 10, social responder)

Similarly, an anonymous participant in the online social responder survey commented:

I have been in this field for a long time (25 years) and with my lived experience have most of this covered, but I really value the way Insight Exchange honor the victim survivor story in the way family violence is described.

For many victim-survivor participants, engaging with Insight Exchange inspired personal and professional ambitions to engage in domestic, family, and sexualized violence advocacy and prevention work. In the process of engaging with Insight Exchange, many victim-survivors also came to recognize their own potential to act as social responders and identified that their lived expertise could meaningfully contribute to the prevention and elimination of violence and abuse. For example, Interviewee 6, said that sharing her lived experience with Insight Exchange initiated her own work as a social responder in the field of domestic and family violence prevention. Similarly, Interviewee 15 who initially shared her lived experiences with Insight Exchange, is now employed in a domestic and family violence service. Interviewee 15 said that she draws from her lived experiences and the key Insight Exchange ideas in her professional work with victim-survivors and in her advocacy work for organizational and systemic change.

Interviewee 5 said that Insight Exchange staff inspired her to engage in domestic and family violence advocacy and prevention work. Interviewee 5 decided to introduce Insight Exchange to several organizations in the entertainment industry where she had professional experience and expertise. Interviewee 5 has since supported several entertainment industry organizations in the development of domestic and family violence policies and procedures. For example, Interviewee 5 helped to develop a policy that entitles victim-survivors employed within the entertainment organization to a “$5000 cash up front, unlimited leave with pay.”

### How Insight Exchange Supports Organizational Change and Improves Social Responses:

Most social responders (*N* = 18) who completed the online survey either agreed or strongly agreed that Insight Exchange had changed the way that they respond to domestic, family, and sexualized violence. Approximately half (*N* = 12) of the social responder participants agreed or strongly agreed that Insight Exchange had contributed to significant organizational change. Of the remaining respondents, eight felt that Insight Exchange had somewhat contributed to organizational change and four reported that Insight Exchange had not contributed to organizational change. Social responder participants indicated that Insight Exchange resources, initiatives, and ideas, had been applied in a range of contexts including the domestic and family violence sector and in other contexts such as Aboriginal and Torres Strait Islander communities, faith communities, LGBTIQA+ communities, Culturally and Linguistically Diverse communities, financial services, corporate sector, arts industry, legal, academic, Men's Behavior Change, education sector, health sector and housing and child protection sectors. In the semistructured interviews, many social responder participants described closely studying and implementing the key concepts communicated through Insight Exchange. This suggests that Insight Exchange resources effectively communicate key messages and concepts enabling social responders to deeply implement the ideas in a diverse range of organizational contexts:

Even though my work does not specifically focus on domestic violence, I can see how the resistance to violence and that journey-mapping had application across all different victim crime types. (Interviewee 8, social responder)

While half of the social responder participants in the online survey did not believe that Insight Exchange had significantly contributed to organizational change, many social responders who participated in the semistructured interviews felt that Insight Exchange had influenced both the organizations they work for and the broader systems and discourses. For instance, some social responders felt that Insight Exchange resources helped increase the capacity for social responders to respond empathically to victim-survivors by understanding their resistance coupled with the understanding that “she is actually the expert in managing her own safety and for her children every day…” (Interviewee 9, social responder). Social responders also described how they used Insight Exchange and Response-Based Practice ideas to educate other social responders during interagency advocacy work. For example, Interviewee 14, regularly introduces coworkers and interagency partners to Insight Exchange and response-based practice ideas to depathologize and decriminalize victim-survivors’ responses and resistance to violence and abuse:

I am thinking of the ‘complex clients’ that nobody seems to be able to work with because they are people who have been resisting in responding to violence for a very long time. Once (a worker) can flip that mentality and see the survivors’ behaviors as ‘it is just a resistance in a response to violence’ and what you need to do is highlight that and work with what is happening in their choices. (Workers) are like, ‘oh, okay.’ Suddenly it is much simpler work. They are not dealing with ‘challenging behaviors’. They are dealing with a person who is trying to survive. So, reframing a lot of that has really helped in that advocacy work …. No longer getting the criminal judgments, but more getting a human element and response, which is what you want. Seeing the human underneath, the circumstances rather than the (victim-survivors) behaviors. (Interviewee 14, social responder)

Interviewee 14 went on to indicate that revealing victim-survivors’ resistance to violence with interagency partners has been instrumental in advocating for victim-survivors to maintain custody of their children and intervening effectively against the perpetrators’ use of systems abuse. For social responders outside of the domestic and family violence sector, the work of Insight Exchange has also helped to redefine some of the core assumptions concerning what domestic and family violence is and how to respond to victim-survivors and perpetrators. Interviewee 4 said that she presented Insight Exchange ideas to executive staff at a higher education institution and convinced executive staff that they had a responsibility to provide workplace support to staff experiencing domestic, family, and sexualized violence. Prior to Interviewee 4's advocacy, workplace supports for victim-survivors was extremely limited:

I had middle age, White males just sitting back in their seats, and executive members, sitting up and just going, 'oh my God, I never realized …'. They were totally engaged. (Interviewee 4, social responder)

Several social responders indicated that Insight Exchange has influenced organizational practices by continuously modeling how to amplify the lived experiences and expertise of victim-survivors:

I think that (Insight Exchange) are good at holding people accountable to being victim-survivor led. I think that it is really easy to fall into the trap as organizations and institutions of advocating or doing policy work and saying 'this is needed, that is needed' … I think Insight Exchange are really good at keeping victims of violence's voice at the forefront. (Interviewee 10, social responder)

Looking at Insight Exchange and looking at that representation of victims’ voices has certainly made me very keen to think about how we use victim voices in designing health care systems that are more (patient focused). (Interviewee 12, social responder)

Other social responders said that they had implemented ideas from Insight Exchange in direct ways. For example, Interviewee 11 reported that the domestic and family violence service she works at uses a routine screening tool (the DVSAT) along with additional questions informed by Insight Exchange and Response-Based Practice:

(Insight Exchange) has given language to the values that we have …. So, it has really shaped our work …. We ask different questions. Like, ‘who else knows about this’ and ‘has it gotten better or worse for you since you spoke to someone’. Or if a woman chooses to tell some of her story, we will ask more questions …. We have seen some increasing disclosures of strangulation. So, now we ask, ‘has there been more strangulation’. When they say ‘he pushed me’, we ask, ‘where are his hands and what did you do when he tried to push you? Where were you? What actions did you take?’. (Interviewee 11, social responder)

Similarly, Interviewee 8, advocated for and successfully implemented Insight Exchange resources in training modules in a government department specializing in the delivery of services to victim-survivors of domestic, family, and sexualized violence. Interviewee 8 described how the previous training modules in the government department, were not effective in educating social responders on how to provide supportive responses to victim-survivors. For Interviewee 8, Insight Exchange resources supported new social responders to confidently implement the ideas of Insight Exchange and Response-Based Practice.

### Trust in Insight Exchange

Most social responder and victim-survivor participants described feeling a high level of trust toward Insight Exchange as an organization and toward specific Insight Exchange staff, resources, and initiatives. As previously mentioned, many social responders with lived experiences of violence and victim-survivors’ felt that they could trust Insight Exchange due to the organization's attention to promoting victim-survivors’ insights and lived experiences:

(Insight Exchange) is authentic. It is people's voice I feel like Insight Exchange has done some of that work for us. So, (Insight Exchange) has captured people's stories. They have captured them in a way that people are comfortable with, and with the purpose of sharing and using and acknowledging. (Interviewee 12, social responder)

Social responder and victim-survivor participants also identified that trust was generated through the perceived quality of research. Some social responders commented that the partnership between the Center for Response-Based Practice and Insight Exchange has been crucial in developing and establishing trusting Insight Exchange resources, initiatives, and ideas. For some social responders, the high level of trust in Insight Exchange resources was evident in the widespread organizational use of the resources and ideas. For example, Interviewee 3, described using Insight Exchange resources with victim-survivors who attended a domestic and family violence service to facilitate conversations and trust:

If a person is shut-down and time is of the essence, I might just run the (Insight Exchange) animation, just so that people can sit with their own thoughts and feelings before they share what they want to share …. It is actually just a really good springboard of reflection for our clients. (Interviewee 3, social responder)

Many social responders and victim-survivors felt that the interpersonal communication with the director and associate director of Insight Exchange deepened their trust in the Insight Exchange initiative:

The director of Insight Exchange was so beautiful, she just opened herself up and said you know, ‘come in and meet me and we will see what we can do’ …. So, I met her, and I was amazed by what she does in this field and her passion for it. (Interview 5, victim-survivor)

Some social responders reported being able to implement Insight Exchange ideas into services and organizations due in part to the development of a trusted partnership, mentor–mentee relationship with the director of Insight Exchange:

I learned so much from the director of Insight Exchange in that process as well. I would go to her with, ‘this is what I want to do. What is your thoughts on how we can get this lived experience narrative across here?’ When I had issues in the workplace, she would always chew the fat with me until we could work through it. (Interviewee 4, social responder)

For many social responders and victim-survivors, trust in Insight Exchange also increased their confidence in implementing Insight Exchange and Response-Based Practice ideas and approaches. For example, Interviewee 2, said that Insight Exchange and Response-Based Practice have become the preferred practice approaches over other models in the direct work with victim-survivors and the preferred model of practice to train social work students:

(Insight Exchange/Response-Based Practice) makes me more confident in how I am responding and also makes me a better supervisor. I feel I am making an impact and my social work students are not just going out there with a bit of patriarchal, individual responsibility model. (Interviewee 2, social responder)

Other social responders commented on Insight Exchange's inclusive and nonjudgmental approach as being important in generating trust and engagement. Interviewee 12 indicated that Insight Exchange creates engagement with social responders who are new to the concepts and approaches without shaming existing practice models:

The specific community-facing (Insight Exchange) resources are really inclusive — it does not mean that you were doing things wrong before. They really acknowledge that people are trying to come at things from the right place. (Interviewee 12, social responder)

### Victim-Survivors as Experts in Their Own Context and Experiences Therefore They Are Best Placed to Guide Decisions About Their Safety

Many social responders described in detail how the work of Insight Exchange has increased their capacity to be victim-survivor led. Social responders said that Insight Exchange helped them to understand that being victim-survivor-led means choosing to be less reactionary, listening to victim-survivors’ accounts of resistance, refusing to impose assumptions, and resisting the impulse to involve police and other first responder services without the leadership, active and ongoing consent of the victim-survivor:

We have got this lovely algorithm, haven't we, if someone's unsafe and you call the police and all the services come into place and everything's better? And clearly, we know, that that does not always work … I think being able to have a level of comfort with listening to people's stories and it is about being able to work with people working out what the next step is rather than having that knee-jerk reaction ‘we need to call the police’, ‘you need to go to hospital’, ‘we need to do this, that and the other’. (Interviewee 12, social responder)

Social responders felt that Insight Exchange had helped them to work holistically with victim-survivors taking into account the victim-survivors’ expertise and their unique situational context. Social responders also described feeling confident in following the choices and leadership of victim-survivors, which results in victim-survivor-led practice rather than crisis-driven practice:

When (victim-survivors) come to us we do not need to jump into crisis because they have been managing their situation and their safety and the safety of their children for years, potentially before connecting with us. So, it is a way of operating that is genuinely client-centered, that respects the agency of women to make choices for themselves, but also, it was a way to keep our staff aligned to the values so that they do not get that 'savior complex'. We do not make victim-survivors prove it and making them tell you the whole story and take away their choice. (Interviewee 11, social responder)

It was a lightbulb moment … every decision we made came back to how we followed the lead of the individual in setting up all of our policies and processes and systems. So really for me, it was that life-changing. (Interviewee 4, social responder)

You know, she might feel that she can manage her risks and safety to a much higher level being in the home versus leaving the home. (Interviewee 9, social responder)

Interviewees 11 and 12 described using Insight Exchange resources to inform and educate coworkers and interagency partners to encourage them to stop responding to victim-survivors’ disclosures with reactivity. By reducing reactivity and increasing the capacity of social responders to sit with “uncomfortable” feelings (Interviewee 12, social responder), social responders felt better equipped to be victim-survivor led. Interviewee 12 gave the example of supporting a doctor, who was extremely concerned about a patient’s safety and wanted to involve the police. Interviewee 12, informed by Insight Exchange supported the doctor to be victim-survivor led and to resist her impulse to involve police without the direction and consent of the victim-survivor:

I was trying to (help the doctor) really understand that the amount of safety that this woman had built … and what she felt as safety and understood by safety, was not necessarily a police version of safety. I was trying to hold both the woman involved and the doctor involved. The doctor was clearly highly engaged with this patient and very supportive. But could not hear the story of what the woman was going through at that time, without feeling that she had to report to the police. And just trying to support them both, in really very legitimately experiencing what they are experiencing. (Interviewee 12, social responder)

Similarly, Interviewee 14, used Insight Exchange and Response-Based Practice ideas to advocate for a victim-survivor who had her baby removed from her custody. Interviewee 14, organized a meeting with child protection staff and explored the victim's-survivor's history and ‘case-notes’ focusing on the victim's-survivor's resistance to her partner’s violence and abuse:

I got the (child protection staff) to sequence it. I said, ‘let us look at this in great detail’ and then they went, ‘oh, my gosh’—they could not see this before. Because there was a woman who had a 3-week-old child removed from her because the partner had set up a situation and accused her of something which did not occur. He was an incredibly dangerous person who had perpetrated violence against a number of other people. But child protection and police only saw (the victim-survivor) in one little instance, and they removed the child from her. They placed the child with the (perpetrator) who was actually quite dangerous …. This woman rang up for help and no one wanted to help her because they saw the initial police report. But I said ‘no, let us look at the greater detail of the circumstance'. (Interviewee, 14, social responder)

Interviewee 14 further explained that once the child protection staff and police understood the victim's-survivor's responses in the context of resistance to violence and once the perpetrator's pattern of violent behavior toward the victim-survivor and others were revealed, the victim's-survivor's child was returned to the victim's-survivor's custody. Interviewee 14, further reflected on the benefit of listening to victim-survivors and reading case notes through the lens of their resistance to violence and how this has the potential for organizational and systemic change focused on upholding the dignity, choice, and control of victim-survivors:

Once you have one or two of those examples you can shift a worker, then you shift the organization. So, (the child protection team) are now doing a lot more work in that space of the complexity of violence, and the complexity of resistance to violence than they historically did. (Interviewee, 14, social responder)

### Limitations in Insight Exchange and Quality Improvement Recommendations

Overwhelmingly, both victim-survivor and social responder participants valued the work of Insight Exchange. There were only very few comments from participants about the limitations of Insight Exchange. Most participants shared their ideas and observations for quality improvement with the hope that Insight Exchange might reach more victim-survivors, influence more social responders, and contribute to national and global domestic, family, and sexualized violence prevention. A few social responders said that Insight Exchange could further support social responders who are utilizing all the introductory Response-Based Practice concepts in their work through ongoing professional development such as the provision of advanced formal courses and/or ongoing supervision. Another social responder felt that Insight Exchange could produce an instructive implementation guide to better support organizations to implement the ideas and approaches, and to foster organizational change. Interviewee 11 acknowledged that the organizational implementation guide would need to be detailed “it is not as simple as X, Y, Z, here is your one-page document” (Interviewee 11, social responder). Similarly, a victim-survivor participant also suggested that Insight Exchange develop an organizational audit tool so that organizations, social responders, and victim-survivors could audit to what extent the organization utilizes the ideas from Insight Exchange and response-based practice. Some social responders and one victim-survivor participant felt that the Insight Exchange resources were too academic and text-heavy, and as a result, would not likely reach victim-survivors and perpetrators who have not had access to higher education or had constrained literacy. Similarly, Interviewee 14 felt that Insight Exchange resources could be more accessible. Interviewee 14 said that in her experience victim-survivors engaged with the Insight Exchange concepts better when she took the time to read out aloud or talk about Insight Exchange resources such as My Safety Kit. Similarly, some victim-survivors said they were less likely to engage with the written resource published in booklet format and preferred audio-visual resources.

## Conclusion

This qualitative study sought to explore how the Insight Exchange initiative may have contributed to the understanding and quality of responses from social responders and how the social responses from Insight Exchange staff were experienced by victim-survivors. The findings from this research project indicate that the work of Insight Exchange has contributed to improving the quality of social responses to victim-survivors of domestic, family, and sexualized violence. To the best knowledge of the authors, this study is the first to explore the Insight Exchange initiative and also contributes to the emerging literature on the application of Response-Based Practice ideas.

There were several methodological limitations to the current study. The recruitment strategy only invited people who were already engaged with Insight Exchange to participate. It is likely that people who signed up to receive email updates from Insight Exchange and people who searched the Insight Exchange website and found the recruitment webpage would have positive associations with the organization in comparison with people who did not take these steps. As such, participants with positive experiences of Insight Exchange may have been overrepresented in the sample of participants. This study was exploratory, and time and resource constraints did not allow for a comprehensive evaluation of the Insight Exchange initiative. Future research could further measure the short and long-term outcomes for victim-survivors of domestic, family, and sexualized violence who engage with organizations that have implemented ideas from Insight Exchange and Response-Based Practice.

This study provides evidence indicating that Insight Exchange improved social responders and victim-survivors’ understanding of domestic, family, and sexualized violence by demonstrating the importance of clear and accurate representations of violence. Social responder participants demonstrated an understanding of how (mis)representations of violence and abuse can influence the quality of responses to victim-survivors, perpetrators, and other social responders. This research study indicates that social responders felt supported by Insight Exchange to use language focused on victim-survivors’ responses and resistance to violence and their efforts to maintain safety and dignity for themselves and others. Social responder participants reported that this focus in conjunction with an analysis of the social/situational context of where and when the abuse took place, supported victim-survivors to make detailed disclosures about the abuse they survived. Social responder participants working in frontline domestic and family violence services reported using the Insight Exchange and Response-Based Practice interview questions during intake to support victim-survivors to make more detailed disclosures while maintaining the safety and dignity oft victim-survivors through focusing on their resistance and responses to violence and abuse. Crucially, the Insight Exchange initiative supported social responders to be victim-survivor led in their responses. Victim-survivor-led approaches prioritizes victim-survivors’ choice and control in making decisions. This approach also minimizes the real or perceived coercion of victim-survivors to make decisions based on the needs of the responder or organization (Wheildon et al., 2022).

Social responder participants reported that they used ideas from Insight Exchange and Response-Based Practice ideas in interagency advocacy work. By revealing victim-survivors’ resistance and responses to violence with interagency partners, one social responder was able to assist a victim-survivor to maintain custody of her children and prevent ongoing systems abuse. This finding is also supported by the work of Alexander et al. (2022) who reported that the social workers/child protection workers who were trained in response-based practice felt attuned to mothers and understood the actions taken by mothers through the lens of responding to and resisting violence. These understandings were found to “enhance the parent–practitioner relationship” and built trust with victim-survivors (Alexander et al., 2022, p. 3595). Furthermore, Alexander et al. (2022, p. 3593) reported that the practitioner use of Response-Based Practice questions changed the thinking of practitioners who tended to “assess the mother as cooperative and protective and were less likely to assess the children as unsafe and in need of protection compared to practitioners” who did not utilize this approach.

Several victim-survivor participants described the social responses they received from Insight Exchange staff members as having a transformational and “life-changing” influence. For many victim-survivors, speaking about their lived experiences of domestic, family, and sexualized violence with Insight Exchange staff was the first time they understood their responses in the context of resisting and responding to violence, and how their choices and strategies had increased safety and dignity for themselves and others. This finding is also supported by Catherine Richardson Kinewesquao et al. (2021, p. 9), who argue that through “Response-Based framework analysis, one can accurately depict violence and mistreatment, which can have a transformational effect on the process of healing.” For victim-survivors who engaged with Insight Exchange, the supportive social responses they received from Insight Exchange staff and associates facilitated some victim-survivor participants to let go of victim-blaming narratives and feelings of shame, and self-blame and increased their ability to resist unhelpful labels. Victim-survivor participants reported that these new insights improved their self-perception, well-being, and their quality of life which supported victim-survivors to work in professional domestic and family violence prevention and advocacy roles. Similarly, social responder participants working directly with victim-survivors, reported that victim-survivors appeared to have greater confidence and well-being when staff implemented the ideas from Insight Exchange and Response-Based Practice in comparison with other approaches.

The success of the Insight Exchange initiative can be strongly attributed to centering the lived experiences of victim-survivors in conjunction with the knowledge translation work of Response-Based Practice ideas. The work of Insight Exchange offers a promising and accessible model to improve social, institutional, and systemic responses to victim-survivors both within and beyond the formal service system. The Insight Exchange/Response-Based Practice ideas can also be applied to other violence prevention contexts for example improving responses to and reducing abuse against; LGBTIQA+ communities, culturally and racialized communities, refugee and new migrant communities, people with lived experience of incarceration, children and youth, elders and older people, people with disabilities. These ideas could also meaningfully contribute to cultural safety and anti-racism, decolonization, and human rights work and practices more broadly.

## Appendix A

### Social Responders’ Perspectives of Insight Exchange—Semistructured In-depth Interview Guide

*Please note that this guide only represents the main themes to be discussed with the participants and as such does not include the various prompts that may also be used (examples given for each question). Nonleading and general prompts will also be used, such as “Can you please tell me a little bit more about that?” and “What does that look like for you?”*

1. How have you engaged in Insight Exchange?
2. How (if at all) have Insight Exchange initiatives/resources contributed to your thinking about domestic and family violence?
3. What is something that you have been reflecting on since your engagement in the Insight Exchange initiative?
4. Has your engagement in Insight Exchange further informed your understanding of domestic and violence?
5. Has your engagement in Insight Exchange changed your understanding of the experiences of victims of violence?
6. Has your engagement in Insight Exchange further contributed to how you understand the behaviors of perpetrators of violence?
7. Has your engagement in Insight Exchange changed how you understand your role as a responder?
8. What (if anything) has changed in how you respond to people who are/were victims of DFV?
9. What (if anything) has changed in how you respond to people who have/are perpetrating violence?
10. If you work with perpetrators directly or in men's behavior change, what are you noticing about they are responding to you following your engagement with Insight Exchange resources? What (if anything) has changed in how your organization/community responds to perpetrators of DFV?
11. How (if at all) have you/your organization/community used Insight Exchange resources?
12. Is there anything else you would like to share regarding your engagement with the Insight Exchange initiative?

## Appendix B

### Victim-Survivors—Semistructured In-depth Interview Guide

1. When you became aware of the opportunity to share your experiences through Insight Exchange, what were your hopes for participating?
2. Looking back, to what extent (if at all) did your participation in an Insight Exchange interview meet your hopes?
3. How would you describe your experience of participating in Insight Exchange interview?
  - What stands out for you about that experience?
  - Can you talk a little bit about your experience of:
    - Participating in the first interview?
    - Participating in the second interview?
    - Reviewing/deidentifying your narrative?
    - Engaging with the Insight Exchange team?
    - Communicating with the team?
4. How did you respond to the Insight Exchange interviewers?
5. What was your overall experience of participating in the Insight Exchange interview?
6. Was any aspect of participating in Insight Exchange helpful?
7. Was any aspect of participation unhelpful or harmful?
8. Have you had further insights about domestic and family violence (not about your own experiences) since your participation in Insight Exchange?
9. Is there anything else you would like to share regarding your experience of participating in the Insight Exchange initiative?

## References

[bibr1-10778012231186812] Alexander K. Humphreys C. Wise S. Zhou A. (2022). Bringing dignity to the assessment of safety for children who live with violence. The British Journal of Social Work, 52(6), 3578–3598. 10.1093/bjsw/bcab260

[bibr2-10778012231186812] Allen N. E. Larsen S. Trotter J. Sullivan C. M. (2013). Exploring the core service delivery of an evidence-based community advocacy program for women with abusive partners. Journal of Community Psychology, 41(1), 1–18. 10.1002/jcop.21502

[bibr3-10778012231186812] Bazeley P. (2009). Analyzing qualitative data: More than identifying themes. Malaysian Journal of Qualitative Research, 2(2), 6–22.

[bibr4-10778012231186812] Bazeley P. (2012). Integrative analysis strategies for mixed data sources. The American Behavioral Scientist (Beverly Hills), 56(6), 814–828. 10.1177/0002764211426330

[bibr5-10778012231186812] Carter N Bryant-Lukosius D Dicenso A Blythe J Neville A J (2014). The Use of Triangulation in Qualitative Research. Oncology Nursing Forum, 41(5), 545–547. 10.1188/14.ONF.545-547 25158659

[bibr6-10778012231186812] Coates L. Wade A. (2004). Telling it like it isn’t: Obscuring perpetrator responsibility for violent crime. Discourse & Society, 15(5), 499–526. 10.1177/0957926504045031

[bibr7-10778012231186812] Coates L. Wade A. (2007). Language and violence; analysis of four discursive operations. Journal of Family Violence, 22(7), 511–522. 10.1007/s10896-007-9082-2

[bibr8-10778012231186812] Coates L. Wade A. (2016). ‘We’re in the 21st century after all’: Analysis of social responses in individual support and institutional reform’. In Hydén M. Gadd D. Wade A. (Eds.), Response-based practice approaches to the study of interpersonal violence (pp. 176–195). Springer.

[bibr9-10778012231186812] Cramp K. Zufferey C. (2021). The removal of children in domestic violence: Widening service provider perspectives. Affilia Journal for Women and Social Work, 36(3), 406–425. 10.1177/0886109920954422

[bibr10-10778012231186812] Domestic Violence Victoria and University of Melbourne (2020). The Family Violence Experts by Experience Framework: Research Report and Framework. Retrieved from https://safeandequal.org.au/wp-content/uploads/DVV_EBE-Framework- Report.pdf

[bibr11-10778012231186812] Donovan A M Dubrasky D Sorensen S Corser G (2019). Poetic license to write resistance: women resisting intimate partner violence through poetry. Critical and Radical Social Work, 7(2), 155–171. 10.1332/204986019X15567130270699

[bibr12-10778012231186812] Fast E Richardson Kinewesquao C (2019). Victim-Blaming and the Crisis of Representation in the Violence Prevention Field. International Journal of Child, Youth and Family Studies, 10(1), 3–25. 10.18357/ijcyfs101201918804

[bibr13-10778012231186812] Fetters M Curry L Creswell J (2013). Achieving Integration in Mixed Methods Designs-Principles and Practices. Health Services Research, 48(6pt2), 2134–2156. 10.1111/1475-6773.12117 24279835 PMC4097839

[bibr14-10778012231186812] Hydén M Wade A Gadd D (2015). Response Based Approaches to the Study of Interpersonal Violence. Palgrave Macmillan UK. https://doi.org/10.1057/9781137409546

[bibr15-10778012231186812] Insight Exchange. (2022). *Insight Exchange*. https://www.insightexchange.net/.

[bibr16-10778012231186812] Insight Exchange. (2022). *Quality response continuum*. In possession of the author.

[bibr17-10778012231186812] Laing L Humphries C (2013). Social Work and Domestic Violence: Developing critical and reflective practice. Sage Publications.

[bibr18-10778012231186812] MacFarlane J. (2020). Going public: A survivor’s journey from grief to action. BTL Books.

[bibr19-10778012231186812] Noonan P. Taylor A. Burke J. (2017). Links between alcohol consumption and domestic and sexual violence against women: Key findings and future directions. ANROWS. https://www.anrows.org.au/publication/links-between-alcohol-consumption-and-domestic-and-sexual-violence-against-women-key-findings-and-future-directions/ .

[bibr20-10778012231186812] NSW Domestic Violence Death Review Team. (2020). A report of the domestic violence death review team pursuant to section 101J (1) of the Coroners Act 2009 (NSW). NSW Government. https://coroners.nsw.gov.au/documents/reports/2017-2019_DVDRT_Report.pdf .

[bibr21-10778012231186812] Overstreet N M Willie T C Sullivan T P (2019). Stigmatizing Reactions Versus General Negative Reactions to Partner Violence Disclosure as Predictors of Avoidance Coping and Depression. Journal of Interpersonal Violence, 34(8), 1734–1752. 10.1177/0886260516653753 27296052 PMC5754257

[bibr22-10778012231186812] Rawsthorne M. (2009). Just like other families? Supporting lesbian-parented families. Australian Social Work, 62(1), 45–60. 10.1080/03124070802626885

[bibr23-10778012231186812] Richardson C. Aviles-Betel K. Ismail-Allouche Z. Picard V. (2021). Healing and rebalancing in the aftermath of colonial violence: An indigenous-informed, response-based approach. Genealogy, 5(3), 69. 10.3390/genealogy5030069

[bibr24-10778012231186812] Richardson C. Wade A. (2015). Taking resistance seriously: A response-based practice approach to social work in cases of violence against indigenous women. In Esquao S. A. Strega S. (Eds.), Walking this path together: anti-racist and anti-oppressive child welfare practice (pp. 204–217). Fernwood Publishing.

[bibr25-10778012231186812] Santos K. d. S. Ribeiro M. C. Queiroga D. E. U. d. Silva I. A. P. d. Ferreira S. M. S. (2020). The use of multiple triangulations as a validation strategy in a qualitative study. Ciência & Saude Coletiva, 25(2), 655–664. 10.1590/1413-81232020252.12302018 32022205

[bibr26-10778012231186812] Sylaska K. M. Edwards K. M. (2014). Disclosure of intimate partner violence to informal social support network members: A review of the literature. Trauma, Violence & Abuse, 15(1), 3–21. 10.1177/1524838013496335 23887351

[bibr27-10778012231186812] Tseris E. (2014). *Diagnosing distress? psychiatric and therapeutic constructions of “Traumatised” young women* [Doctoral thesis]. The Sydney eScholarship Repository. http://hdl.handle.net/2123/13479

[bibr28-10778012231186812] Ursa M. Koehn C. (2015). Young women’s experiences of coping with violence in intimate relationships. Journal of Mental Health Counseling, 37(3), 250–267. 10.17744/1040-2861-37.3.250

[bibr29-10778012231186812] Wheildon L. J. True J. Flynn A. Wild A. (2022). The batty effect: Victim-survivors and domestic and family violence policy change. Violence Against Women, 28(6-7), 1684–1707. 10.1177/10778012211024266 34431729

[bibr30-10778012231186812] Wilson J. Goodman L. A. (2021). “A community of survivors”: A grounded theory of organizational support for survivor-advocates in domestic violence agencies. Violence Against Women, 27(14), 2664–2686. 10.1177/107780122098114 33529567

[bibr31-10778012231186812] Wilson M. (2022). Understanding refusals, using coercion: Young men’s understanding and use of normalized sexualized violence within heterosex. The Journal of Sex Research, Ahead-of-Print(Ahead-of-Print), 1–13. 10.1080/00224499.2022.2086676 35723597

